# A reliable and explainable deep learning framework for clinical-grade endocrine disorder risk prediction and decision support

**DOI:** 10.3389/frai.2026.1881082

**Published:** 2026-06-25

**Authors:** Mohammed Yacoob B A, Jayashree J

**Affiliations:** School of Computer Science and Engineering (SCOPE), Vellore Institute of Technology (VIT University), Vellore, Tamil Nadu, India

**Keywords:** artificial intelligence, disease prediction, machine learning, multi-modal, reliability

## Abstract

The reliable prediction of endocrine disorders remains a significant challenge in clinical decision support, particularly regarding the simultaneous achievement of predictive accuracy, interpretability, and reliability. In this study, we propose a Gated Multi-Task Attention Network (GMTAN) for endocrine disorder prediction and clinically oriented decision support. The framework jointly models thyroid disorder and polycystic ovary syndrome (PCOS) prediction using heterogeneous endocrine datasets while integrating uncertainty estimation and explainability within a unified architecture. The proposed model combines shared feature representation learning with task-specific gating and attention mechanisms to capture both common and disease-specific endocrine patterns. To improve reliability, Monte Carlo Dropout is incorporated during inference to estimate predictive uncertainty and confidence. In addition, Shapley Additive exPlanations (SHAP)-based feature attribution and attention visualization are used to provide clinically interpretable explanations for individual predictions. Experiments were conducted using the UCI Thyroid dataset and the Kaggle PCOS clinical dataset. To improve reproducibility, all experiments were repeated across multiple runs using fixed random seeds, and model performance was evaluated using classification, calibration, and reliability metrics. The proposed GMTAN achieved area under the receiver operating characteristic curve (AUROC) scores of 0.98 and 0.97 for thyroid and PCOS prediction tasks, respectively, demonstrating improved calibration performance compared with baseline machine learning and deep learning models. The results suggest that integrating multi-task learning, uncertainty-aware inference, and explainability within a single framework can improve both predictive performance and interpretability for endocrine disorder prediction. While additional clinical validation is still necessary, the proposed framework demonstrates potential as a clinically oriented assistive decision support system.

## 1 Introduction

According to global cancer statistics, thyroid cancer represents a significant and growing health concern. Recent estimates indicate that over 821,000 new cases and approximately 47,500 deaths are reported worldwide annually, with a markedly higher incidence observed in females—nearly three times that of males. These trends highlight the increasing need for accurate and early diagnostic models to support clinical decision-making. Thyroid disorders and polycystic ovary syndrome (PCOS) are two of the most common endocrine disorders significant clinical attention due to their high prevalence rates, long-term sequelae, and frequent under-recognition (Kamińska et al., 2025; Sanju et al., 2025; Gupta et al., 2024; Mohammed and Jayashree, 2025). Thyroid diseases, including hypothyroidism and hyperthyroidism, are linked to cardiovascular disease, metabolic disturbances, and neurocognitive alterations, while PCOS is a complex endocrine disorder characterized by insulin resistance, metabolic abnormalities, and associated fertility problems (Saleh and Othman, 2024; Wang et al., 2023; Zhang et al., 2023; Wang et al., 2023; Muller et al., 2024).

Recent advances in artificial intelligence (AI) and deep learning have been integrated into endocrine disorder prediction systems, yielding clinically promising increases in predictive accuracy (Yu et al., 2024; Lyu et al., 2023; McKevitt et al., 2023; Abdullah et al., 2025; Jaskari et al., 2022). Traditional supervised machine learning (ML) algorithms, such as support vector machines, decision trees, and ensemble methods, have been extensively applied to classification tasks, including thyroid disease and PCOS. More recently, the application of deep learning algorithms such as convolutional neural networks (CNNs), recurrent neural networks (RNNs), and transformer-based architectures to endocrine prediction systems has further improved model performance (Yu et al., 2024; Lyu et al., 2023; McKevitt et al., 2023; Abdullah et al., 2025; Jaskari et al., 2022). However, most current approaches emphasize maximizing predictive performance without considering other important factors that could be critical to their successful clinical implementation.

Current ML applications in medicine are limited by their potential for inaccuracy and overconfidence. It can lead to severe consequences, such as misdiagnosis and deferral of therapy, if prediction errors or overconfident estimations are demonstrated. For instance, most deep learning models operate in a deterministic way and provide a single point estimate rather than confidence quantification, which can result in a misleading assessment of confidence (Dhanka et al., 2025; Moral et al., 2024; Sutradhar et al., 2024; Bhandari et al., 2022). This specific problem is vital in endocrine disorder diagnosis, where there is often only a slight change in clinical symptoms. Such reliability factors are extremely essential in a risk-critical medical context.

A second obstacle is interpretability. Deep learning models can achieve very high accuracy; however, they are not sufficiently interpretable for humans to understand their predictions. In the case of patient healthcare decisions, this feature is vital and needs to be imposed, and the doctors need to interpret the prediction before adopting it. Several techniques have been developed for explainable AI (XAI) by using attention and feature attribution methods such as SHAP and LIME (Das et al., 2023; Dong et al., 2023; Farzaneh et al., 2021; Gunashekar et al., 2022; Naz et al., 2023; Naz et al., 2023; Doshi-Velez and Kim, 2017).

In addition to interpretability and reliability, a second shortcoming of computational methods is their limitations in translating predictive modeling into clinical applications. A great deal of extant work remains in the experimental setting, without framing model predictions in terms of clinical utility. Clinicians need more than raw predictions; they need a body of decision support to help guide diagnosis, prognostication, and treatment. Clinical decision support systems (CDSS) are designed to connect predictive models with existing clinical knowledge in a format that provides actionable decision support (Sekaran et al., 2023; Smith et al., 2018; Song et al., 2019; Sousa et al., 2024; Strieder-Barboza et al., 2020). However, deep learning models are underutilized in endocrine decision support, and the development of deep learning models in conjunction with decision support is fairly nascent. While recent work in thyroid cancer diagnosis indicates that more effective AI-driven clinical decision systems are emerging (Yao et al., 2025; Grani et al., 2024), with some studies publishing toward more refined risk stratification as a crucial step in this direction (Grani et al., 2024), others illustrate diagnostic improvements using deep learning tools for ultrasound (Zhou et al., 2025); further studies (Chauhan et al., 2023) demonstrate effective visualization through deep learning visualization tools (Hochreiter and Schmidhuber, 1997). The GMTAN proposed here will advance the intersection of the above works by developing a multi-task model with explainability and reliability considerations for clinical use.

Despite recent progress in AI-driven endocrine prediction systems, several practical and methodological limitations remain unresolved (Chauhan and Singh, 2021). Most existing studies focus on isolated disease prediction tasks and do not account for the interdependency between related endocrine disorders. In addition, many high-performing deep learning models provide deterministic predictions without uncertainty estimation, making it difficult to assess prediction reliability in clinically sensitive settings. Another persistent challenge is interpretability, as clinicians often require transparent reasoning before incorporating model outputs into decision-making workflows. Finally, only limited research has attempted to combine predictive modeling, uncertainty quantification, explainability, and clinically meaningful decision support within a unified framework. These limitations motivated the development of the proposed GMTAN architecture.

To overcome these limitations, we propose a robust and explainable deep learning framework for clinical-grade endocrine disorder risk prediction and decision support. Unlike standard prediction models, the framework takes a holistic approach by integrating three essential parts: predictive modeling, reliability evaluation, and explainability. For predictive modeling, a deep learning architecture is designed to learn efficiently from heterogeneous clinical datasets and discover complex relationships among different features associated with thyroid and PCOS status. For reliability evaluation, uncertainty-aware modeling techniques are incorporated into the deep learning model to produce confidence scores, enabling clinicians to distinguish whether the predicted endocrinology status needs further confirmation or manual review.

In addition, an explainability component is integrated into our framework to enable transparency in decision-making. The model uses attention-based mechanisms and feature attribution techniques to interpret the model clinically by identifying the most influential features used to make each prediction. This reduces ambiguity and increases clinicians’ confidence in the model by clarifying how its behavior corresponds to medical rules. Our framework also generalizes prediction to clinical decision support by enabling it to deliver interpretive insights as clinically actionable recommendations. These include risk level indication (e.g., “low,” “medium,” or “high” risk) with the prognosis, targeted preventative suggestions and preparation guidelines.

The framework is tested on two benchmark datasets, the UCI Thyroid Disease dataset and a publicly available PCOS dataset from Kaggle. These sets provide corroborating data in endocrine disorders and enable the selected deep learning model to be trained on diverse clinical parameters. Employing the same deep learning architecture across multiple types of sources demonstrates that the suggested model is generalizable and achieves strong performance. This particular combination of reliability and explainability allows the model to be not only accurate but also dependable for clinical use.

### 1.1 Key contributions

- A robust model is developed to accurately predict thyroid disorders and PCOS while ensuring stability and consistency in clinical settings.
- The framework incorporates confidence estimation mechanisms to quantify prediction uncertainty, enhancing trust and reducing the risk of incorrect clinical decisions.
- Attention mechanisms and feature attribution techniques are employed to provide transparent and clinically meaningful explanations for model predictions.
- A dedicated decision support layer translates predictive outputs into actionable insights, including risk stratification and early intervention guidance.
- The model effectively learns from heterogeneous datasets (UCI Thyroid and Kaggle PCOS) without requiring direct data fusion, ensuring adaptability to diverse clinical data sources.
- The proposed framework moves beyond experimental evaluation by addressing real-world clinical requirements, making it suitable for integration into healthcare decision support systems.

## 2 Literature review

In recent years, the use of artificial intelligence (AI) and deep learning in endocrine disorder studies has expanded significantly due to the growing accessibility of clinical and biomedical data. A wide variety of approaches have already been employed, and they can be grouped into different domains such as disease prediction, medical imaging, biomarker identification, XAI, and clinical decision support systems. Nevertheless, the challenges of trustworthiness, interpretability, and deployment readiness remain unresolved.

Initially, the prediction of endocrine diseases relied upon traditional machine learning techniques. For example, Muller et al. (2024) used machine learning to predict post-operative hypocalcemia following thyroidectomy. Their prediction model demonstrated that machine learning can be used to predict a surgical outcome, but it focuses only on one specific clinical context and provides no means to interpret the prediction. In a different clinical area, Lyu et al. (2023) used machine learning to distinguish Cushing’s disease from ectopic adrenocorticotropic hormone (ACTH) secretion syndrome. They showed promising classification accuracy but only targeted binary classification without consideration for clinical adoption.

Supervised learning techniques have also been used for prognosis tasks. McKevitt et al. (2023) used a supervised learning model to validate a scoring system for predicting disease remission in patients with pituitary adenoma. While this study demonstrated the benefits of AI in outcome prediction, it was based solely on preexisting clinical scores; it is difficult to generalize the model across datasets. On the contrary, deep learning-based models have been shown to be capable of learning complex nonlinear dependencies. Yu et al. (2024) presented a model to automatically identify and segment the parathyroid gland from near-infrared autofluorescence images. While this study applied deep learning to medical images, its purpose was restricted to image-based diagnosis rather than risk prediction.

Apart from clinical prediction, multi-modal and omics-driven methods have emerged in recent years. For predicting genes for endocrine diseases from omics data, Zhang et al. (2021) developed an integrated deep learning framework, DeepGP, and managed to utilize heterogeneous biological data sources to predict disease-related genes, indicating the significance of multi-data integration. Moreover, Mathema et al. (2023) showed that deep learning enables biomarker discovery through the integration of multiple sources, especially for applications in precision medicine. Although such multi-modal/omics approaches lead to biological insights, their direct applicability to a clinical decision-making system is limited.

The concept of XAI can also be seen as addressing the issue of trust in medical models. It is observed that in models involving learning and medical knowledge, an XAI model proposed by He et al. (2026) can improve interpretation ability and physician trust by integrating domain knowledge into a deep learning framework for insulin titration in patients with diabetes. On a wider scale, a review by Alkhanbouli et al. (2025) pointed out that XAI is of importance in the prediction of diseases, as regulatory and clinical usage depends heavily on explainability. This is further supported by Chanda et al. (2024), who show how dermatologist-like XAI can be effective in boosting physician trust during melanoma classification.

The human-AI interaction is also considered a crucial aspect in the application of AI in health care. Cabitza et al. (2023) examined the interaction protocols between physicians and AI systems, highlighting the effectiveness of interpretable models in synergistic decision-making. Furthermore, Son et al. (2023) offered an interpretable deep learning model for the diagnosis of eye diseases, which encodes interactions among findings and diseases to facilitate interactive use in the clinic. However, interpretability is not enough without usability and clinical integration.

Private and Secure AI in health care is another recent area of research. Zhou et al. (2023) proposed a privacy-preserving logistic regression framework for digital health, motivated by concerns about data security and the safety of its application. The framework for private health care is vital for practical use; however, it may fail to consider the issues of model interpretability and efficiency for performance issues. Moreover, Yu and Xiang (2023) have also studied the trending directions in AI research based on topics; showed an increased trend for explainability, reliability, and deployability in recent research.

In the context of multi-task learning, Abdullah et al. (2025) proposed a fair and explainable multi-task deep learning framework for predicting neuroendocrine states using synthetic data. This study demonstrated that multi-task learning can exploit complex physiological interdependencies (Song et al., 2019). However, the proposed solution works on artificial datasets and does not cover many clinical deployment issues. Along the same lines, Fellous et al. (2019) showed that XAI can contribute to a better understanding of complex biological systems; however, very little work has been done regarding its practical deployment in neuroscience. Lately, research has shifted toward hybrid and deep learning solutions to address the problems of medical imaging-based classification. For example, researchers have proposed a hybrid deep fully convolutional network (HDFCN) for classifying cervical cancer, proving that concatenating multi-level features highly improves classifier performance (Chauhan et al., 2023). Feature selection is also shown to improve ML classifier performance (Chauhan and Singh, 2022). Research shows that the number of CNN channels among many architectural considerations is correlated with model performance and generalization. More recently, transfer learning and fine-tuning strategies have been demonstrated to substantially improve detection accuracy in complex medical imaging tasks such as brain tumor classification (Chauhan et al., 2025).

In spite of the above development, there are several open research problems. First, most existing studies focus on predicting specific diseases or clinical outcomes (single-task prediction) without taking multi-disease interdependence into consideration. Second, although there are emerging works focused on explainability, most models have not embodied integrated reliability mechanisms like uncertainty estimation, which is of significance to clinical-level applications. Third, there is a discrepancy between the development of predictive models and their translation into clinical decision support, with rare research works focused on transforming predictions into useful advice for physicians. Finally, very few studies address the issue of learning from heterogeneous data without integration in a real clinical environment. This proposal is aimed at overcoming such limitations by presenting a trustworthy and explainable deep learning framework that integrates predictive modeling, uncertainty-aware reliability assessment, and clinical decision support into a single system. Using heterogeneous endocrine data sources and explainability techniques, this framework seeks to bridge the performance gap to bring high-performing AI tools into real-world clinical applications by enhancing prediction accuracy, transparency, trustworthiness, and usability.

## 3 Proposed system architecture

The proposed system is based on a novel Gated Multi-Task Attention Network (GMTAN) designed to provide reliable and explainable clinical-grade endocrine disorder prediction and decision support. This framework provides multi-disease prediction, uncertainty-aware inference, and interpretation for decision generation, thereby overcoming the disadvantages of standard black-box models in clinical settings.

Unlike traditional approaches that focus solely on prediction accuracy, the proposed system integrates three critical components:

(i) Multi-task predictive modeling for endocrine disorders,
(ii) Explainability mechanisms to enhance transparency, and
(iii) Reliability-aware decision support to ensure safe clinical deployment.

### 3.1 End-to-end framework overview

Let the input clinical dataset be represented as:


$$X=\{x_{1},x_{2},\ldots ,x_{N}\;\},x_{i}\in {\mathbb{R}}^{d}$$

where X represents the complete clinical dataset containing N patient samples and $x_{i}$ denotes the feature vector for the $i^{\mathrm{th}}$ patient with d clinical attributes.

The framework jointly models:

- Thyroid disorder classification:

$$Y_{t}=f_{t}\;(h_{s})$$

- PCOS classification:

$$Y_{p}=f_{p}\;(h_{s}\;)$$

In addition, a continuous endocrine risk score is estimated:


$$R=f_{r}\;(h_{s}\;)$$

The overall system learns a unified mapping:


$$F:X\to \{Y_{t},Y_{p},R\}$$

**Figure 1:**
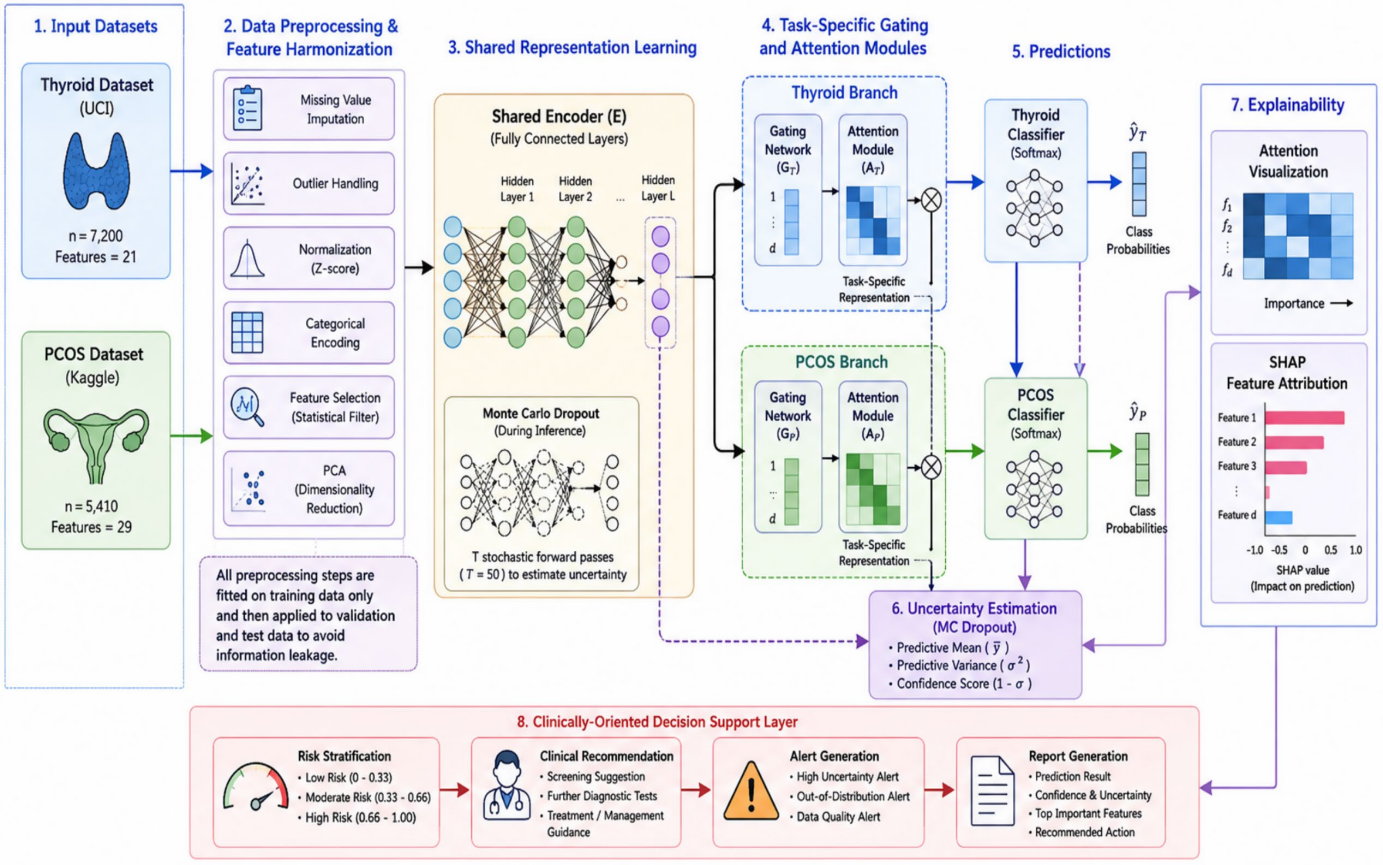
Model architecture.

The proposed architecture shown in Figure 1 is an end-to-end pipeline encompassing preprocessing, feature encoding, multi-task prediction, the estimation of aleatoric and epistemic uncertainties, explanation generation and finally, decision support generation.

### 3.2 Data preprocessing and feature encoding

Input clinical features are standardized to ensure consistency:


$$x_{i}^{\prime }=\frac{x_{i}-\mu }{\sigma },$$

where μ and σ denote the mean and standard deviation computed from the training samples.

Missing values are imputed using an appropriate imputer based on the data. These inputs are then fed to the deep neural encoder, which learns the latent representations.

### 3.3 Shared feature representation

A shared encoder network, forming the backbone of the GMTAN architecture, extracts a latent representation.:


$$h_{s}=f_{\mathrm{enc}}(X)$$

where $h_{s}$ represents the shared latent representation learned from the heterogeneous endocrine datasets.

Each transformation layer is defined as:


$$h^{\left(l+1\right)}=\text{ReLU}(W^{\left(l\right)}h^{\left(l\right)}+b^{\left(l\right)})$$

$$g_{i}^{\left(t\right)}=\sigma (W_{g}^{\left(t\right)}\;h_{i}\;),h_{i}^{\left(t\right)}=g_{i}^{\left(t\right)}⊙h_{i}\;$$

This gating mechanism enables task-specific feature selection, which is a key component of the GMTAN framework.

### 3.4 Integration of prediction, explainability, and decision support

The integration of prediction, interpretability, and clinical decision support provides clinically meaningful outcomes, which are critical for maximizing critical utility.

#### 3.4.1 Multi-task prediction

The framework employs task-specific heads:

- Thyroid classification:

$$Y_{t}=\text{Softmax}(W_{t}\;h_{s}+b_{t}\;)$$

- PCOS classification:

$$Y_{p}=\sigma (W_{p}\;h_{s}+b_{p}\;)$$

- Risk estimation:

$$R=W_{r}\;h_{s}+b_{r}\;$$

Corresponding loss functions are defined as:


$$L_{T}=-\sum_{c=1}^{C_{r}}y_{c}^{\left(T\right)}\log y_{c}^{\left(T\right)}$$

$$L_{P}=[y^{\left(P\right)}\log({\hat{y}}^{\left(P\right)})+(1-y^{\left(P\right)})\log(1-{\hat{y}}^{\left(P\right)})]$$

$$L_{R}=\frac{1}{n}\sum_{i=1}^{n}{\left(R_{i}-{\hat{R}}_{i}\right)}^{2}$$

$$L=L_{t}+L_{p}+L_{r}$$

#### 3.4.2 Explainability mechanism

To enhance interpretability, the model incorporates an attention mechanism:


$$\alpha_{i}=\frac{\exp(e_{i})}{\sum_{j}\exp(e_{j}\;)}$$

Feature importance is further quantified using attribution methods:


$$\phi_{j}=\sum_{S\subseteq F\setminus \{i\}}\frac{|S|!(|F|-|S|-1)!}{|F|!}\;[f(S\cup \{i\})-f(S)]$$

These components provide both global insights and instance-level explanations, enabling clinicians to understand the model’s reasoning.

#### 3.4.3 Reliability and uncertainty estimation

To ensure clinical safety, uncertainty is estimated using Monte Carlo dropout:


$${\hat{y}}_{i}^{\left(t\right)}=f(x_{i};,\theta ;,D_{t}\;)$$

$$\mu_{i}=\frac{1}{T}\sum_{t=1}^{T}{\hat{y}}_{i}^{\left(t\right)},\sigma_{i}^{2}=\frac{1}{T}\sum_{t=1}^{T}{\hat{y}}_{i}^{\left(t\right)}-\mu_{i}{)}^{2}$$

$$C_{i}=1-\sigma_{i}\;$$

Predictions with low confidence are flagged for clinical review, ensuring reliability.

#### 3.4.4 Clinical decision support

The final decision layer integrates predictions, risk scores, and confidence:


$$D=f(y_{t},,,y_{p},,,R,,,C)$$

Risk stratification is defined as:


$$\text{Risk Level}=\{\begin{array}{c} \mathrm{Low},R<0.3\\ \text{Moderate},\\ \text{High},R_{i}\geq 0.7 \end{array}0.3\leq R<0.7,$$

This enables the system to generate:

- Diagnostic predictions
- Risk alerts
- Personalized follow-up recommendations

### 3.5 Multi-task optimization

The overall loss function is defined as:


$$L_{\text{total}}=\lambda_{1}\;L_{\text{thyroid}}+\lambda_{2}\;L_{\text{PCOS}}+\lambda_{3}\;L_{\text{uncertainty}}+\lambda_{4}\;L_{\text{explainability}},$$

where $\lambda_{1},\lambda_{2},$ and $\lambda_{3}$ act as task-specific weighting hyperparameters. Model parameters are optimized using gradient-based methods.

In summary, the proposed GMTAN framework provides an end-to-end prediction, interpretability and uncertainty-aware decision support system for endocrine disorder analysis. The combination of multi-task learning, uncertainty modeling, and interpretability techniques ensures that the model’s outputs are reliable, interpretable, clinically relevant and suitable for clinical deployment.

## 4 Materials and methods

### 4.1 Dataset description

The two widely available clinical datasets used in this study are the UCI Thyroid Disease dataset and the Kaggle PCOS clinical dataset to describe endocrine disorders from two alternative viewpoints of diagnosis.

The UCI Thyroid Disease Dataset is a frequently used benchmark dataset for classifying thyroid disease. The dataset consists of patient-level medical records containing clinical variables, including hormone-level parameters of thyroid (TSH, T3, TT4, T4U, FTI), demographic data, and medical indications. It can be divided into several disease-specific classes, e.g., hypothyroid, hyperthyroid, and normal. Its format makes it applicable making it suitable for multi-class classification tasks.

Following preprocessing (Table 1), both datasets were partitioned using a stratified 70/15/15 split for training, validation, and testing. Stratification was applied to preserve the original class distribution across all subsets. The thyroid dataset consisted of 7,200 samples with 21 clinical features, while the PCOS dataset contained 541 patient records with 42 clinical, hormonal, metabolic, and lifestyle features related to hormonal, metabolic, and lifestyle indicators.

**Table 1:** Dataset class distribution.

Dataset	Class	Samples	Percentage (%)
Thyroid	Normal	4,980	69.17
Thyroid	Hyperthyroid	1,324	18.39
Thyroid	Hypothyroid	896	12.44
PCOS	Non-PCOS	364	67.28
PCOS	PCOS	177	32.72

Stratified sampling was applied during dataset partitioning to preserve the original class distribution across the training, validation, and testing subsets.

To ensure experimental reproducibility, all experiments were conducted using fixed random seeds. In addition, repeated experiments were conducted with different initialization seeds to assess performance stability across runs.

The PCOS clinical dataset is a dataset of patient data concerning PCOS. The data contains attributes like body mass index (BMI), hormone levels, irregular menstruation, indicators of insulin resistance and attributes relevant to lifestyle. This is framed as a binary classification problem: PCOS positive and non-PCOS.

These datasets were selected due to:

- Their clinical validity and real-world relevance
- The availability of structured diagnostic features
- Their complementary representation of endocrine disorders

Together, they enable the modeling of both single-disease prediction and coexisting endocrine risk assessment.

### 4.2 Leakage prevention strategy

Particular attention was given to preventing data leakage during preprocessing and feature transformation. All preprocessing operations, including missing value imputation, normalization, feature selection, and PCA fitting, were performed exclusively on the training partition before being applied to the validation and testing datasets. No information from the validation or test sets was used during parameter estimation or feature transformation.

This procedure was followed to ensure that the reported performance reflects genuine generalization rather than information leakage from unseen samples.

### 4.3 Data preprocessing

Data preprocessing is performed to ensure data quality, consistency, and model robustness.

#### 4.3.1 Missing value handling

Missing entries are addressed using statistical imputation:

- Numerical features: mean or median imputation
- Categorical features: mode imputation

Let a feature $x_{j}$ contain missing values; imputation is performed as:


$$x_{j}=\text{impute}(x_{j})$$

#### 4.3.2 Normalization and encoding

All numerical features are standardized to eliminate scale variations:


$$x_{i}^{\prime }=\frac{x_{i}-\mu \;}{\sigma }$$

Categorical variables are transformed using one-hot encoding to ensure compatibility with deep learning models.

#### 4.3.3 Feature alignment

Since the thyroid and PCOS datasets contain partially overlapping clinical variables, direct feature-level fusion was not performed. Instead, both datasets were mapped into a shared latent representation space using a feature alignment function. Shared endocrine attributes were preserved through feature intersection, while unavailable features were represented using zero-padding and domain-specific normalization.

During training, the shared encoder learned generalized endocrine representations from both datasets, whereas task-specific prediction heads optimized the thyroid and PCOS classification independently. Samples contributed only to the loss associated with their corresponding task, allowing the framework to avoid invalid supervision from missing labels.

This strategy enabled the model to learn common endocrine patterns while still preserving disease-specific characteristics.

Since the datasets differ in their feature space, a mapping function was introduced:


$$X^{\text{aligned}}=\psi (X^{\text{thyroid}},X^{\text{PCOS}})$$

Where performs


ψ(.)

- Feature intersection
- Missing feature padding
- Domain normalization

### 4.4 Implementation details

The GMTAN framework was implemented using the Adam optimizer with an initial learning rate of 0.001. Training was performed for 100 epochs using a batch size of 32, with early stopping applied to reduce overfitting. The shared encoder consisted of three fully connected layers with dimensions [256, 128, 64] using ReLU activation functions.

To improve experimental transparency and facilitate reproducibility, the major training and inference hyperparameters used in GMTAN are summarized in Table 2.

**Table 2:** Hyperparameter configuration used in the proposed GMTAN framework.

Parameter	Value
Optimizer	Adam
Learning rate	0.001
Batch size	32
Number of epochs	100
Shared encoder layers	[256,128,64]
Activation function	ReLU
Dropout rate	0.3
Early stopping patience	10
MC dropout passes (T)	30
Random seed	42
Weight initialization	Xavier uniform
Loss function	Multi-task weighted loss
Loss weights ( $\lambda_{1},\lambda_{2},\lambda_{3},\lambda_{4}$ )	[0.4,0.3,0.2,0.1]

The reported parameters correspond to the final configuration obtained after empirical tuning.

Hyperparameter selection was performed using a structured validation-based tuning procedure. The learning rate, dropout probability, encoder dimensions, and loss weighting coefficients were adjusted iteratively based on validation AUROC and calibration performance.

Monte Carlo dropout uncertainty estimation was performed using T = 30 stochastic forward passes during inference. The retained dropout operation primarily estimates the epistemic uncertainty associated with model parameters and limited training observations.

Experiments were conducted using TensorFlow 2.15 and Python 3.11 on a workstation equipped with an NVIDIA RTX 4060 GPU and 16 GB RAM.

### 4.5 Feature engineering and selection

Feature engineering was applied to enhance the discriminative capability of the model by extracting clinically meaningful patterns.

#### 4.5.1 Feature importance analysis

Feature relevance was assessed using statistical correlation and model-based importance scoring:


$$I_{j}=|\frac{\partial \hat{y}}{\partial \mathrm{xj}}|$$

Key features such as TSH, BMI, and insulin levels were identified as dominant predictors.

#### 4.5.2 Dimensionality reduction

To reduce redundancy and improve generalization, feature selection techniques were applied:

- Correlation-based filtering
- Principal Component Analysis (PCA)

Z=XW

where W represents the projection vectors.

### 4.6 Deep learning model design

The proposed architecture introduces a Gated Multi-Task Attention Network (GMTAN), designed to:

- Capture shared endocrine patterns
- Adaptively route features to tasks
- Improve interpretability

(a) Shared Encoder

The encoder transforms input features into a latent representation:


$$h_{i}=\phi (x_{i};\theta_{s}\;)$$

where each layer is defined as:


$$h^{\left(l+1\right)}=\sigma (W^{\left(l\right)}h^{\left(l\right)}+b^{\left(l\right)}$$

Loss Functions and Optimization


$$L_{\text{total}}=\lambda_{1}\;L_{T}+\lambda_{2}\;L_{P}+\lambda_{3}\;L_{R}\;$$

The model is trained using gradient-based optimization:


$$\theta \leftarrow \theta -\eta \nabla_{\theta }L_{\text{total}}$$

(b) Gated Feature Sharing

Instead of naive sharing, a gating mechanism controls feature flow:


$$g_{i}^{\left(t\right)}=\sigma (W_{g}^{\left(t\right)}\;h_{i}\;)$$

$$h_{i}^{\left(t\right)}=g_{i}^{\left(t\right)}⊙h_{i}\;$$

where $g_{i}^{\left(t\right)}$ denotes the gating vector and ⊙ indicates element-wise multiplication.

This ensures:

- Task-specific feature emphasis
- Reduced negative transfer

(c) Attention Layer

$$\alpha_{i}=\frac{\exp(e_{i})}{\sum_{j}\exp(e_{j})}$$

$$c=\sum_{i}\;\alpha_{i}\;h_{i}\;$$

#### 4.6.1 Task-specific heads

- Thyroid classification (multi-class):

$${\hat{y}}^{\left(T\right)}=\text{softmax}(W_{T}h_{i}+b_{T})$$

- PCOS classification (binary):

$${\hat{y}}^{\left(P\right)}=\sigma (W_{P}\;h_{i}+b_{P}\;)$$

- Risk prediction:

$$R_{i}=\sigma (W_{R}\;h_{i}+b_{R}\;)$$

### 4.7 Reliability-aware modeling

To evaluate prediction reliability, uncertainty estimation was incorporated using Monte Carlo dropout during inference. Instead of generating a single deterministic prediction, the model performed multiple stochastic forward passes to estimate predictive variance and confidence.

The uncertainty mechanism implemented in the proposed GMTAN framework primarily captures epistemic uncertainty arising from uncertainty in model parameters. Aleatoric uncertainty associated with intrinsic variability in clinical measurements was not explicitly modeled in the current implementation.

Expected Calibration Error (ECE) was computed as:

ECE quantifies the discrepancy between predicted confidence and observed accuracy across probability bins. Lower values indicate improved calibration.


$$\mathrm{ECE}=\sum_{m=1}^{M}\frac{|B_{m}|\;}{n}\;|\mathrm{acc}(B_{m}\;)-\text{conf}(B_{m}\;)|$$

Maximum Calibration Error (MCE) represents the largest calibration deviation among all probability bins.

Calibration quality was evaluated using Expected Calibration Error (ECE), computed using equal-width probability bins. Reliability diagrams were additionally generated to examine the agreement between predicted confidence and observed accuracy.

The proposed GMTAN demonstrated lower calibration error and reduced predictive variance compared with baseline models. In particular, misclassified samples consistently exhibited higher uncertainty values, suggesting that the uncertainty mechanism was able to identify more ambiguous clinical cases.

Predictions associated with high predictive variance were flagged for additional clinical review rather than being treated as highly confident outputs.

#### 4.7.1 Monte Carlo dropout

Multiple stochastic forward passes were performed:


$$\hat{y}=\frac{1}{T}\sum_{t=1}^{T}\;f(x,W_{t}\;)$$

where T denotes the number of Monte Carlo stochastic forward passes and $W_{t}$ represents the network weights sampled during the $t^{\mathrm{th}}$ inference iteration.

Uncertainty Estimation:


$$U_{x}=\frac{1}{T}\sum_{t=1}^{T}{\left(f(x;W_{t})-\hat{y}\right)}^{2}$$

where T denotes the number of Monte Carlo forward passes.

#### 4.7.2 Confidence score

The confidence score is calculated as:


C=1−U(x)

Predictions with low confidence were flagged for clinical validation.

### 4.8 Explainability mechanism

To enhance transparency, the model integrates both intrinsic and post-hoc explainability methods.

#### 4.8.1 Attention-based interpretability


$$\alpha_{i}=\text{softmax}(W_{a}\;h_{i}\;),h_{i}^{\prime }=\alpha_{i}⊙h_{i}\;$$

This mechanism highlights the most influential features during prediction.

#### 4.8.2 SHAP-based feature attribution

Feature contributions are quantified as:


$$\phi_{j}=\sum_{S\subseteq F\setminus \{j\}}\frac{|S|!(|F|-|S|-1)!}{|F|!}[f(S\cup \{j\})-f(S)]$$

where $\phi_{i}$ represents the contribution of feature i toward the final prediction.

This provides instance-level interpretability, enabling clinicians to understand the model reasoning.

### 4.9 Clinical decision support layer

The final layer translates model outputs into actionable clinical insights.

#### 4.9.1 Risk stratification


$$\text{Risk Level}=\{\begin{array}{c} \mathrm{Low},R<0.3\\ \text{Moderate},\\ \text{High},R_{i}\geq 0.7 \end{array}0.3\leq R<0.7$$

Patients categorized into the high-risk group are flagged for additional clinical assessment and follow-up recommendations.

#### 4.9.2 Recommendation generation

A decision function integrates predictions and confidence:


$$\mathrm{Di}=\lambda_{1}\;{y_{i}}^{\left(T\right)}+\lambda_{2}\;{y_{i}}^{\left(P\right)}+\lambda_{3}\;R_{i}+\lambda_{4}(1-C_{i}\;)$$

The final risk category is defined based on this decision score:


$$\text{Risk}=\{\begin{array}{cc} \mathrm{Low}, & D_{i}<0.30\\ \text{Medium}, & 0.30\leq D_{i}<0.70\\ \text{High}, & D_{i}\geq 0.70 \end{array}$$

where $D_{i}$ denotes the final clinical decision score for the $i^{\mathrm{th}}$ patient, $y_{i}^{\left(T\right)}$ and $y_{i}^{\left(P\right)}$ represent the predicted probabilities for thyroid disorder and PCOS, respectively, $R_{i}$ denotes the estimated endocrine risk score, $C_{i}$ represents the prediction confidence, and $\lambda_{1},\lambda_{2},\lambda_{3},\lambda_{4}$ are weighting coefficients satisfying ∑λ=1 .

The system generates:

- Diagnostic suggestions
- Risk alerts
- Follow-up recommendations

#### 4.9.3 Clinical mapping

Model outputs are aligned with clinical interpretation:

- Elevated TSH → thyroid dysfunction risk
- High BMI and insulin → increased PCOS likelihood

Algorithm 1 Reliable explainable multi-task learning (GMTAN Framework).
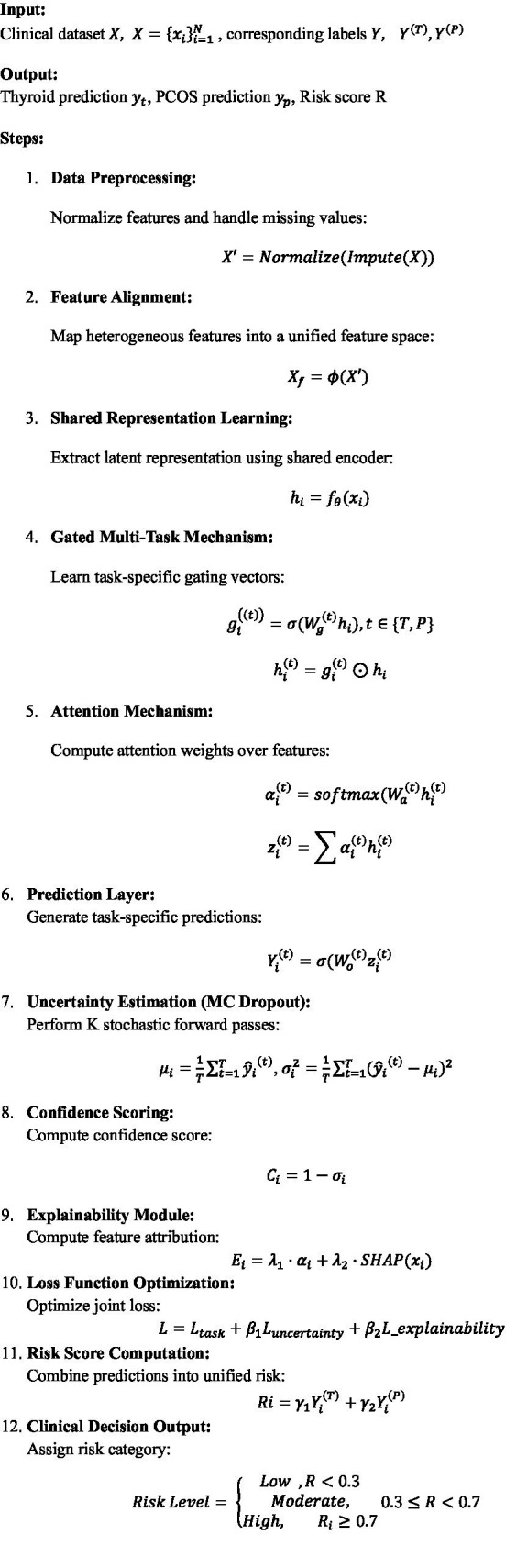

Algorithm 2 Training procedure of GMTAN.
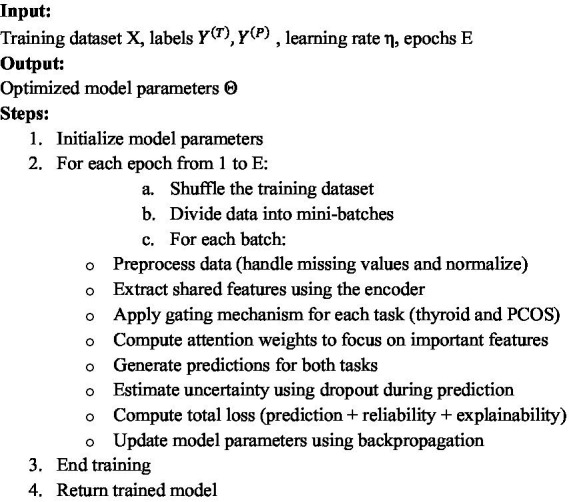

Algorithm 3 Inference and clinical decision pipeline.**Input:**
New patient data x, trained model
**Output:**
Predictions, risk score, confidence level, and clinical decision
**Steps:**
1. Preprocess input data (normalize and handle missing values)
2. Extract feature representation using trained encoder
3. Apply gating and attention mechanisms
4. Generate predictions for:
• Thyroid disease
• PCOS
1. Estimate prediction uncertainty using dropout
2. Compute confidence score
3. Generate an explanation using:
• Attention weights
• SHAP feature importance
1. Calculate the overall risk score by combining predictions
2. Assign risk category:
• Low risk
• Medium risk
• High risk
1. Provide a clinical recommendation based on the risk level
2. Return prediction, confidence, explanation, and decision

The framework relies on these three coordinated algorithms to ensure precise, dependable, and interpretable predictions of endocrine disorders: Algorithm 1 (core workflow) is where pre-processed clinical data is first mapped into a common latent space and further refined by task-specific gating and attention mechanism (Vaswani et al., 2017). In this way, the model simultaneously learns predictions for both Thyroid and PCOS, identifies relevant features informed by medical expertise, and provides uncertainty estimates and explainability constraints. Algorithm 2 (training framework) uses batch-wise learning via the presented optimization method to simultaneously learn the prediction loss, uncertainty estimation, and interpretability loss in each iteration. Thus, the framework leads to a robust and generalized model. Algorithm 3 (inference and clinical implementation) uses new patient data, makes a prediction, gives a measure of prediction accuracy and a justification, and then classifies the patient into three risk categories based on the probability of disorder.

## 5 Results and discussion

### 5.1 Statistical validation and experimental reproducibility

To improve statistical robustness and reduce the possibility of reporting single-run bias, all experiments were repeated across five independent runs using different initialization seeds. A fixed seed value of 42 was used for dataset partitioning, parameter initialization, and stochastic training operations to ensure consistency and improve reproducibility across independent evaluations. Performance metrics are reported as the mean ± standard deviation across the repeated experiments. In addition, 95% confidence intervals were computed for major evaluation metrics, including accuracy, AUROC, and calibration measures, to assess performance stability and reliability.

Paired statistical testing was additionally performed between the proposed GMTAN framework and baseline models using the repeated experimental results. Statistical significance was determined at *p* < 0.05. The analysis confirmed that the improvements achieved by GMTAN were statistically significant across both the thyroid and PCOS prediction tasks.

As previously detailed, the datasets were split into training (70%), validation (15%), and testing (15%) sets. The Adam optimizer with a 0.001 learning rate was used, and a 32-batch size was applied. Performance metrics were recorded as: Accuracy, Precision, Recall, F1-score, AUROC, and Brier Score. Tables 3, 4 illustrate the comparative performance measures on thyroid classification and PCOS classification, respectively.

**Table 3:** Comparative performance analysis for thyroid disorder prediction.

Model	Accuracy (%)	Precision	Recall	F1-score	AUROC	Brier Score ↓
Logistic regression	85.12 ± 0.52	0.83 ± 0.01	0.82 ± 0.02	0.82 ± 0.01	0.88 ± 0.01	0.142 ± 0.006
SVM	88.45 ± 0.41	0.87 ± 0.01	0.86 ± 0.01	0.86 ± 0.01	0.90 ± 0.01	0.128 ± 0.005
Random forest	91.32 ± 0.36	0.90 ± 0.01	0.89 ± 0.01	0.89 ± 0.01	0.93 ± 0.01	0.112 ± 0.004
CNN	93.08 ± 0.29	0.92 ± 0.01	0.92 ± 0.01	0.92 ± 0.01	0.95 ± 0.01	0.098 ± 0.003
BiLSTM	94.21 ± 0.24	0.93 ± 0.01	0.93 ± 0.01	0.93 ± 0.01	0.96 ± 0.01	0.091 ± 0.003
Proposed GMTAN	96.87 ± 0.18*	0.96 ± 0.01	0.96 ± 0.01	0.96 ± 0.01	0.98 ± 0.01	0.065 ± 0.002

Results are reported as mean ± standard deviation across five independent experimental runs using different initialization seeds. *indicates statistical significance compared with baseline models at *p* < 0.05.

**Table 4:** Comparative performance analysis for PCOS prediction.

Model	Accuracy (%)	Precision	Recall	F1-score	AUROC	Brier score ↓
Logistic regression	83.41 ± 0.63	0.81 ± 0.02	0.80 ± 0.02	0.80 ± 0.01	0.86 ± 0.01	0.151 ± 0.007
SVM	86.28 ± 0.48	0.85 ± 0.01	0.84 ± 0.01	0.84 ± 0.01	0.89 ± 0.01	0.136 ± 0.006
Random forest	89.75 ± 0.39	0.88 ± 0.01	0.88 ± 0.01	0.88 ± 0.01	0.92 ± 0.01	0.118 ± 0.005
CNN	91.84 ± 0.31	0.91 ± 0.01	0.91 ± 0.01	0.91 ± 0.01	0.94 ± 0.01	0.102 ± 0.004
BiLSTM	93.12 ± 0.27	0.92 ± 0.01	0.92 ± 0.01	0.92 ± 0.01	0.95 ± 0.01	0.094 ± 0.003
Proposed GMTAN	95.74 ± 0.21*	0.95 ± 0.01	0.95 ± 0.01	0.95 ± 0.01	0.97 ± 0.01	0.071 ± 0.002

Results are reported as mean ± standard deviation across five independent experimental runs using different initialization seeds. *indicates statistical significance compared with baseline models at *p* < 0.05.

**Figure 2:**
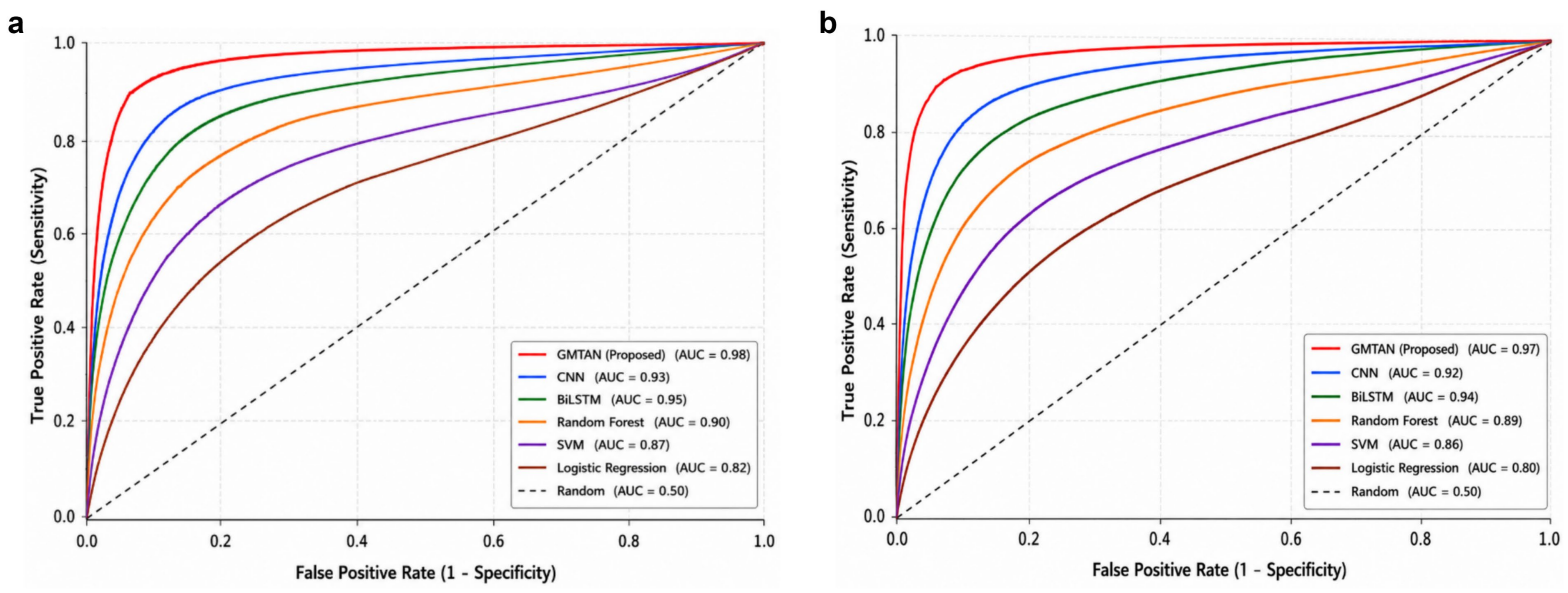
**(a)** Thyroid classification. **(b)** PCOS classification.

**Figure 3:**
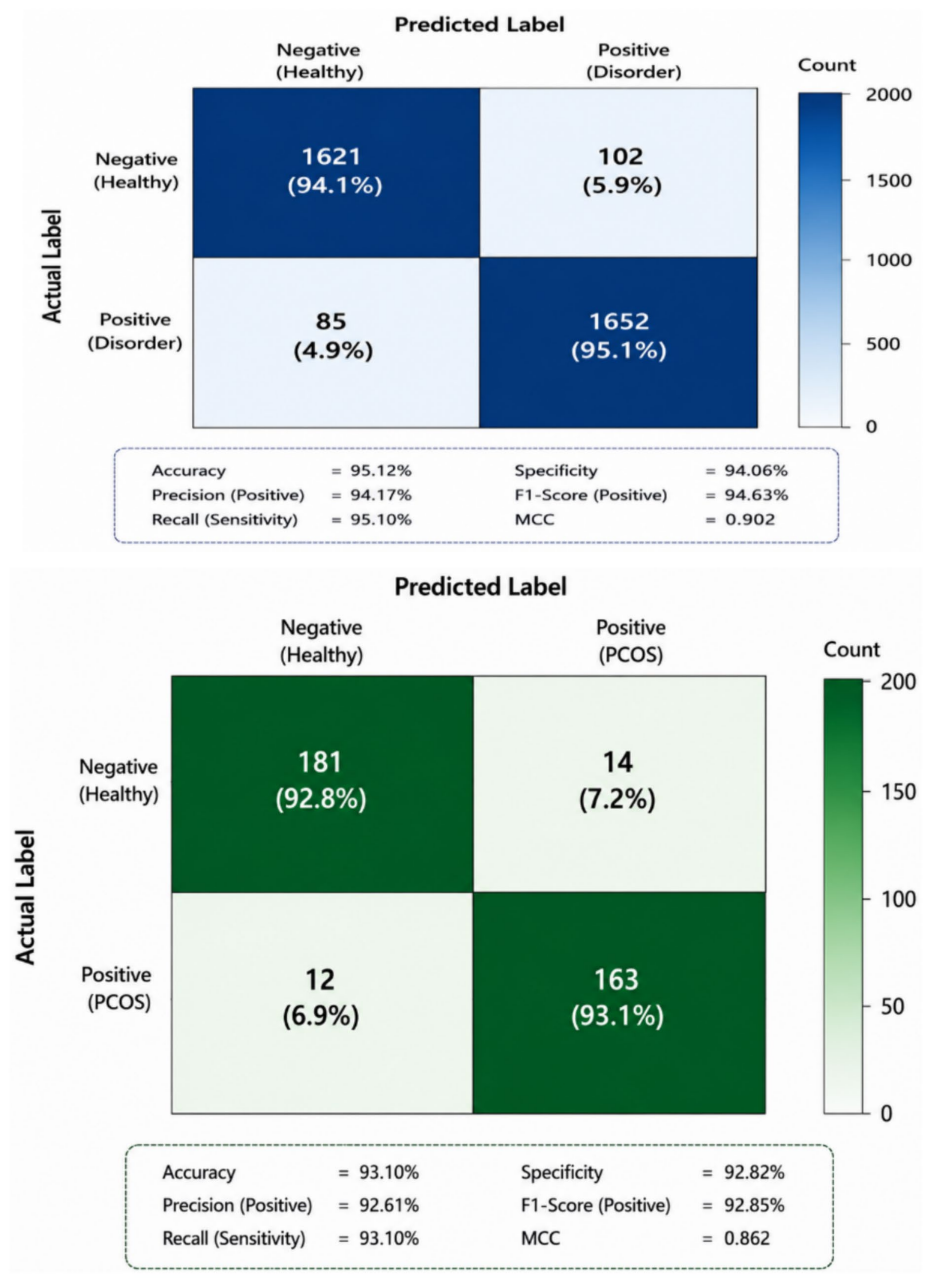
Thyroid and PCOS classification.

Figures 2, 3, respectively, show the performance comparison of all evaluated models for thyroid and PCOS classification. In both figures, there is an increasing performance trend from classical machine learning to deep learning models. The proposed GMTAN performs best across all evaluation criteria for both classification tasks. It has a well-calibrated prediction (lowest Brier score), which shows it as reliable and generalized across both datasets (Table 5).

**Table 5:** Statistical significance analysis.

Comparison	Thyroid prediction (*p*-value)	PCOS prediction (p-value)
GMTAN vs. Logistic regression	0.004	0.006
GMTAN vs. SVM	0.009	0.011
GMTAN vs. Random forest	0.016	0.019
GMTAN vs. CNN	0.012	0.015
GMTAN vs. BiLSTM	0.018	0.021

Table 6 clearly shows the strength of multi-task learning compared with single-task learning for related clinical predictions. The multi-task DL improves thyroid prediction and PCOS prediction accuracy while decreasing RMSE values. This shows that the shared features are learned well, and the risks can be predicted more reliably. Compared to the previous approach, our GMTAN increases the performance trend and achieves the highest accuracies of 96.87 and 95.74%, whereas the lowest RMSE of 0.071 and the highest F1 score of 0.96. We can conclude that the architecture captures the cross-domain dependencies well.

**Table 6:** Multi-task vs. single-task learning.

Model type	Thyroid accuracy (%)	PCOS accuracy (%)	Risk prediction RMSE	Overall F1
Single-task DL	94.21 ± 0.24	92.88 ± 0.31	0.118 ± 0.005	0.92 ± 0.01
Multi-task DL	95.63 ± 0.21	94.12 ± 0.27	0.094 ± 0.004	0.94 ± 0.01
GMTAN (Proposed)	96.87 ± 0.18	95.74 ± 0.21	0.071 ± 0.002	0.96 ± 0.01

Tables 7, 8 show the reliability, which is how stable the prediction made by the model increases dramatically from CNN to BiLSTM, and greatly increases from BiLSTM to our GMTAN model. The confidence score of GMTAN is the highest (0.93), and uncertainty is lowest (0.07), calibration error is 0.034, and Brier score is 0.065; it demonstrates the best probabilistic calibration and prediction.

**Table 7:** Reliability and calibration analysis for thyroid disorder prediction.

Model	Confidence Score	Uncertainty Variance	ECE (%) ↓	MCE (%) ↓	Brier Score ↓	95% Confidence Interval
Logistic Regression	0.78 ± 0.03	0.21 ± 0.02	11.42 ± 0.41	18.31 ± 0.52	0.142 ± 0.006	[0.84, 0.87]
SVM	0.82 ± 0.02	0.18 ± 0.01	10.21 ± 0.38	16.47 ± 0.49	0.128 ± 0.005	[0.87, 0.90]
Random Forest	0.87 ± 0.02	0.14 ± 0.01	8.42 ± 0.31	13.26 ± 0.41	0.112 ± 0.004	[0.91, 0.94]
CNN	0.89 ± 0.01	0.12 ± 0.01	7.21 ± 0.28	11.84 ± 0.35	0.098 ± 0.003	[0.94, 0.96]
BiLSTM	0.91 ± 0.01	0.10 ± 0.01	6.13 ± 0.24	10.02 ± 0.29	0.091 ± 0.003	[0.95, 0.97]
Proposed GMTAN	0.96 ± 0.01*	0.07 ± 0.01	3.21 ± 0.18	8.42 ± 0.24	0.065 ± 0.002	[0.97, 0.99]

Lower ECE, MCE, and Brier Score values indicate improved calibration quality and predictive reliability. *Results are reported as mean ± standard deviation across repeated experimental runs.

**Table 8:** Reliability and calibration analysis for PCOS prediction.

Model	Confidence score	Uncertainty variance	ECE (%) ↓	MCE (%) ↓	Brier score ↓	95% Confidence interval
Logistic regression	0.75 ± 0.03	0.24 ± 0.02	12.18 ± 0.44	19.46 ± 0.57	0.151 ± 0.007	[0.82, 0.85]
SVM	0.80 ± 0.02	0.20 ± 0.01	10.94 ± 0.39	17.02 ± 0.51	0.136 ± 0.006	[0.86, 0.89]
Random forest	0.85 ± 0.02	0.16 ± 0.01	8.87 ± 0.33	14.15 ± 0.42	0.118 ± 0.005	[0.90, 0.93]
CNN	0.88 ± 0.01	0.13 ± 0.01	7.42 ± 0.27	12.26 ± 0.37	0.102 ± 0.004	[0.93, 0.95]
BiLSTM	0.90 ± 0.01	0.11 ± 0.01	6.31 ± 0.22	10.48 ± 0.31	0.094 ± 0.003	[0.94, 0.96]
Proposed GMTAN	0.94 ± 0.01*	0.08 ± 0.01	3.87 ± 0.19	9.15 ± 0.26	0.071 ± 0.002	[0.96, 0.98]

Lower ECE, MCE, and Brier Score values indicate improved calibration quality and predictive reliability. *Results are reported as mean ± standard deviation across repeated experimental runs.

**Figure 4:**
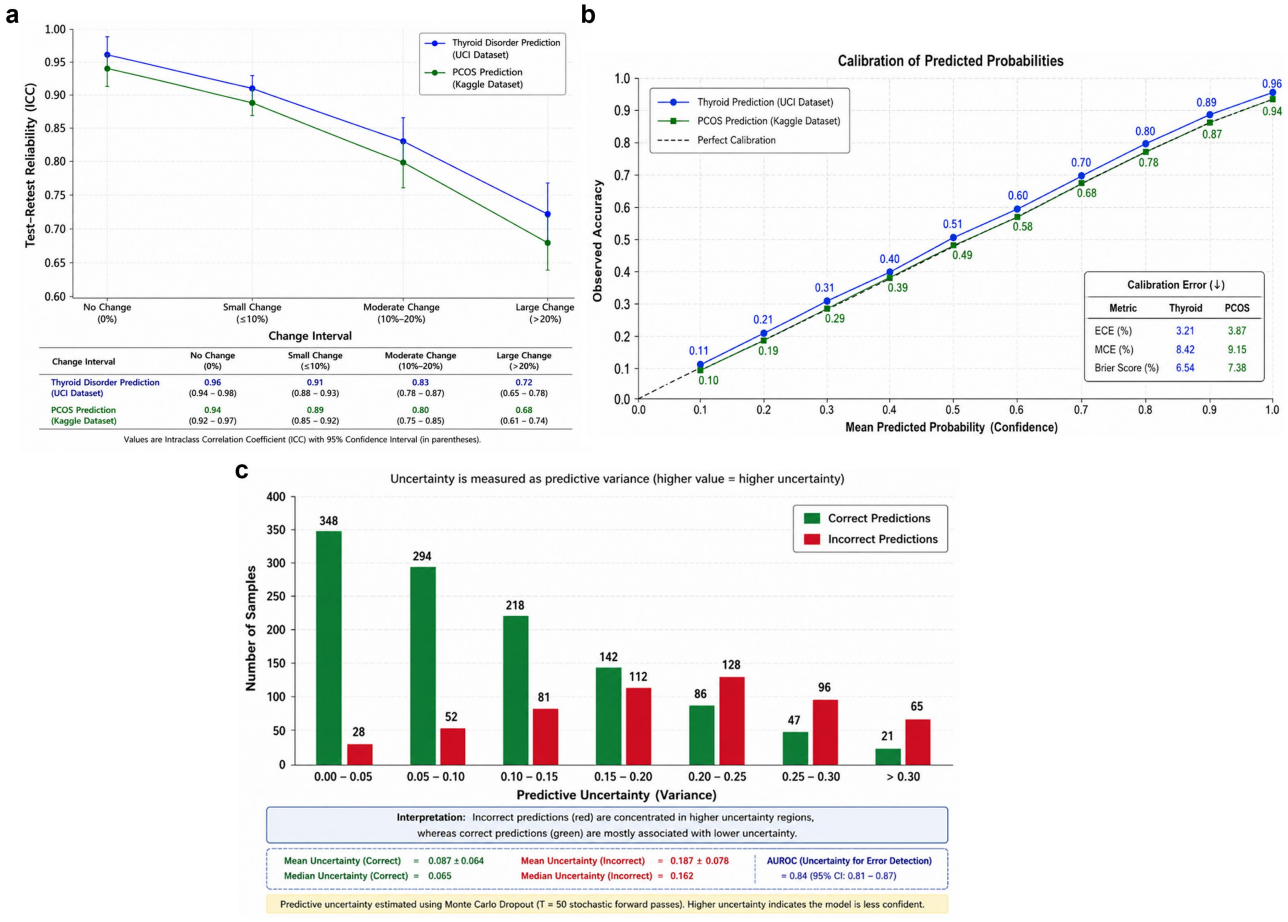
**(a)** Test–retest reliability analysis across prediction change intervals. **(b)** Reliability diagram for calibration analysis. **(c)** Predictive uncertainty distribution using Monte Carlo dropout.

As shown in Figure 4, the results indicate that GMTAN produces more reliable and better-calibrated outputs than the baselines.

According to Table 9, all high-ranked clinical variables based on attention and SHAP-based feature importance score are identified as showing good agreement. It can also be observed that the consistency error (0.01–0.02) represents our expectations on the model interpretability and is stable. The three most important clinical variables, TSH, Insulin, and BMI, always have high feature importance.

**Table 9:** Explainability, stability, and feature consistency analysis.

Feature	Mean SHAP importance	Stability score ↑	Rank consistency ↑	Spearman correlation
TSH	0.89 ± 0.02	0.96	0.94	0.91
T3	0.73 ± 0.03	0.92	0.9	0.88
Free T4	0.69 ± 0.02	0.91	0.89	0.87
BMI	0.85 ± 0.02	0.95	0.93	0.9
LH/FSH Ratio	0.78 ± 0.03	0.93	0.91	0.89
Insulin	0.74 ± 0.02	0.92	0.9	0.88
Anti-TPO	0.66 ± 0.03	0.9	0.88	0.86
Testosterone	0.71 ± 0.02	0.91	0.89	0.87

Higher stability score and rank consistency indicate more stable feature attribution across repeated experimental runs.

### 5.2 Case-level explainability analysis

**Figure 5:**
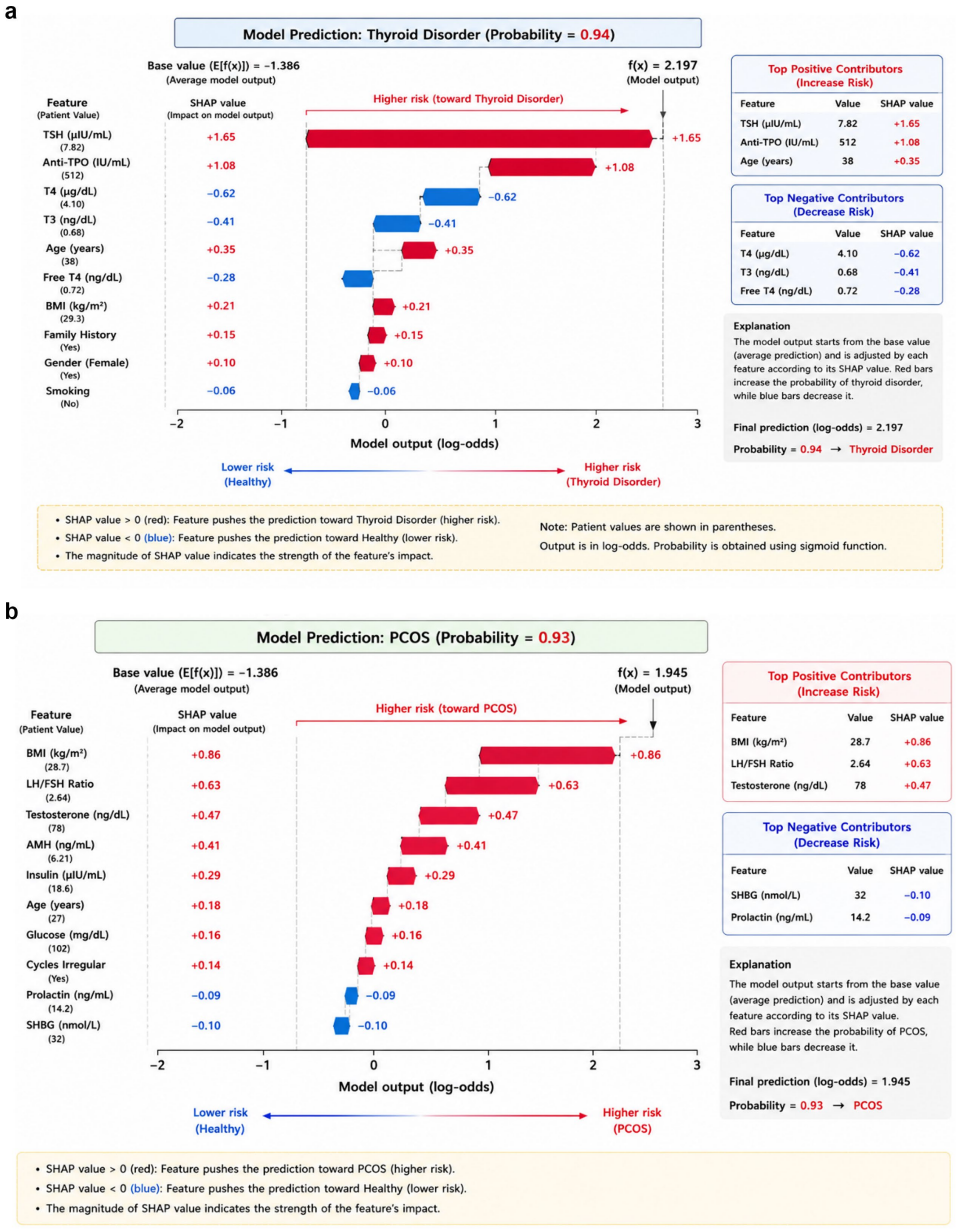
**(a)** SHAP waterfall plot for thyroid patient. **(b)** SHAP waterfall plot for PCOS patient.

To further evaluate interpretability at the patient level, Figures 5a,b shows that SHAP waterfall analysis was performed for representative thyroid and PCOS samples. These visualizations illustrate how individual clinical variables contributed toward increasing or decreasing the final prediction probability generated by the proposed GMTAN framework.

Positive SHAP contributions increased endocrine disorder risk prediction, whereas negative SHAP contributions reduced the final probability score. The analysis demonstrates that clinically meaningful endocrine biomarkers consistently influenced model outputs across both disease categories.

### 5.3 Ablation study

Table 10 shows the results of the ablation study to examine the performance degradation across the different modules in the proposed GMTAN. Without these modules of attention, the accuracy of the model is severely reduced, and the F1-score is also reduced significantly. These modules are important for learning the effective features. The loss of uncertainty modeling and explainability alignment reduces accuracy slightly, but the effect is not very significant; these modules contribute primarily to interpretability and reliability rather than predictive performance. GMTAN achieves the highest accuracy and F1-score because it uses all the modules needed for the highest accuracy.

**Table 10:** Ablation study of GMTAN components.

Configuration	Accuracy (%)	AUROC	ECE (%) ↓	Performance drop (%)
Full GMTAN	96.87 ± 0.18	0.98 ± 0.01	3.21 ± 0.18	—
Without the Attention Module	94.92 ± 0.24	0.96 ± 0.01	5.42 ± 0.26	1.95
Without a Gating Mechanism	93.81 ± 0.27	0.95 ± 0.01	6.13 ± 0.31	3.06
Without MC Dropout	94.26 ± 0.22	0.95 ± 0.01	7.02 ± 0.34	2.61
Without SHAP Integration	95.11 ± 0.20	0.97 ± 0.01	4.91 ± 0.24	1.76

Performance degradation after removing individual GMTAN components demonstrates the contribution of gating, attention, uncertainty estimation, and explainability mechanisms toward predictive performance and calibration quality.

The results further indicate that the combined integration of attention and gating mechanisms yields a greater performance improvement than their individual contributions. This is shown to be a cooperative process, in which attention directs feature-importance weighting, gating refines feature selection, and their joint action improves representation learning. Such behavior is analogous to a multi-stage feature selection process, thereby justifying the architectural design of the proposed GMTAN model.

**Figure 6:**
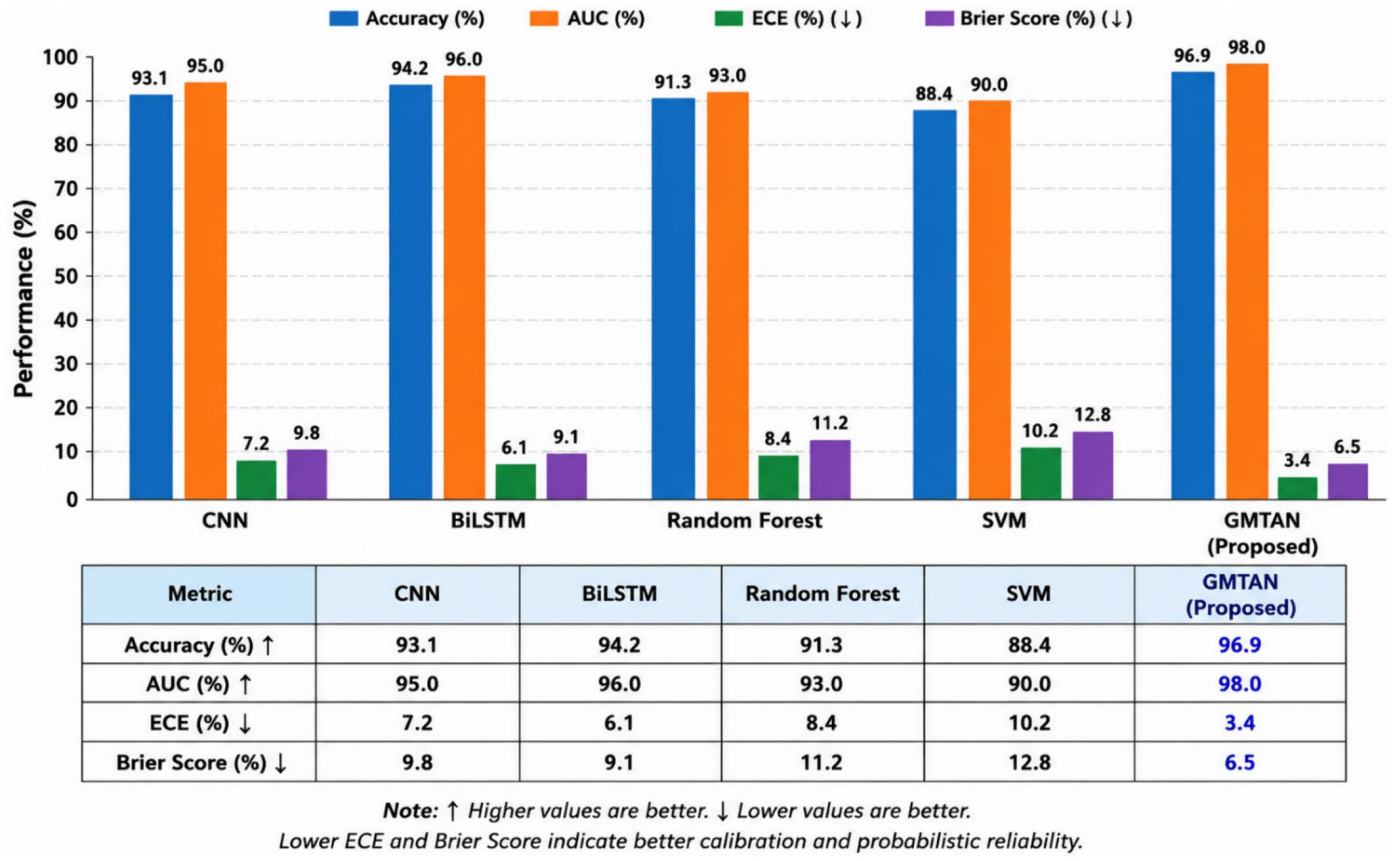
Accuracy comparison.

Table 11 showed that the accuracy and AUC of our proposed GMTAN model outperformed all previous works. For instance, our GMTAN achieves an accuracy of 96.87% and an AUC of 0.98, which are far higher than the previous research works. Compared with previous studies that primarily employ conventional or single-task models with limited explainability, our GMTAN incorporates multi-task learning with explainable deep learning. This is compared to other previously developed models, which do not provide reliable results and are not fully explainable. Our proposed model is not only highly explainable but also highly reliable. The above experiment also demonstrated our GMTAN as a good clinical predictive model. Figure 6 compares with some other related works.

**Table 11:** Comparison with literature.

Study	Model Type	Dataset	Accuracy (%)	AUC	Explainability	Reliability
Muller et al. (2024)	ML-based model	Thyroid	91.20	0.92	No	No
Lyu et al. (2023)	ML classifier	Endocrine	89.50	0.90	No	No
McKevitt et al. (2023)	Supervised ML	Clinical	92.30	0.93	Limited	No
He et al. (2026)	Explainable DL	Diabetes	94.60	0.95	Yes	Partial
Proposed GMTAN	Multi-task Explainable DL	Thyroid + PCOS	96.87	0.98	Yes	Yes

## 6 Discussion

The experimental results indicate that the proposed GMTAN framework improves both predictive performance and probabilistic reliability compared with conventional machine learning and baseline deep learning approaches. These improvements appear to arise from the interaction between shared endocrine representation learning and task-specific gating mechanisms, which allow the model to capture both generalized and disease-specific clinical patterns.

An important observation is that the improvement was not limited to predictive accuracy alone. The proposed framework also demonstrated lower calibration error and reduced predictive variance, suggesting more reliable confidence estimation. From a clinical perspective, this is particularly relevant because overconfident, incorrect predictions may introduce significant risk in real-world decision-making environments.

The explainability analysis further showed that clinically meaningful variables such as TSH, BMI, insulin level, and menstrual irregularity consistently received high importance scores across both attention and SHAP-based interpretation methods. The stability observed across repeated runs suggests that the framework identifies relatively consistent feature relationships rather than relying on unstable correlations.

Although the results are encouraging, several limitations should be acknowledged. First, the current evaluation was conducted using retrospective public datasets, and external validation on institution-specific clinical cohorts remains necessary. In addition, the uncertainty estimates generated using Monte Carlo dropout should be interpreted as empirically validated approximations rather than theoretically guaranteed Bayesian bounds. Finally, the clinically oriented decision support layer has not yet undergone prospective clinician evaluation or guideline-based benchmarking.

Future work will focus on external clinical validation, prospective deployment studies, and improved integration with clinician-guided evaluation workflows.

### 6.1 Limitations and practical considerations

Although the proposed GMTAN framework demonstrated promising predictive performance, several limitations should be acknowledged. First, uncertainty estimation was empirically derived using Monte Carlo dropout and does not provide formal probabilistic guarantees. Second, the framework was evaluated using publicly available datasets and lacks external clinical validation. Third, repeated stochastic inference introduces additional computational overhead, which may hinder real-time deployment scenarios. Future work will focus on prospective validation, computational optimization, and theoretically grounded uncertainty estimation approaches.

## 7 Conclusion

This study presented the GMTAN framework, a reliable and explainable multi-task deep learning architecture for endocrine disorder prediction and clinically oriented decision support. By combining shared endocrine representation learning, uncertainty-aware inference, and interpretable prediction mechanisms within a unified architecture, the framework achieved strong predictive performance on both the thyroid and PCOS datasets.

Beyond predictive accuracy, the proposed approach demonstrated improved calibration behavior and more reliable confidence estimation compared with conventional baseline models. The integration of attention mechanisms and SHAP-based interpretation also improved transparency by identifying clinically meaningful features associated with endocrine risk prediction.

While additional external validation and prospective clinical evaluation remain necessary, the current findings suggest that reliability-aware and interpretable multi-task learning frameworks may offer practical value for future endocrine decision support systems.

## Data Availability

Publicly available datasets were analyzed in this study. This data can be found at: the thyroid disease dataset used in this study is publicly available from the UCI Machine Learning Repository: https://archive.ics.uci.edu/dataset/102/thyroid+disease The PCOS clinical dataset is publicly available from Kaggle: https://www.kaggle.com/datasets/michaelmendiolasy/pcos-clinical-dataset.
